# Hyposulphite of Soda*Read before the Ohio State Veterinary Medical Association:—

**Published:** 1891-03

**Authors:** E. H. Shepherd


THE JOURNAL
OF
COMPARATIVE MEDICINE AND
VETERINARY ARCHIVES.

VOL. XII. MARCH, 1891. NO. 3.

### HYPOSULPHITE OF SODA.*

BY E. H. SHEPHERD, V. S.

We have an agent classed among our veterinary remedies whose virtues are not appreciated as perhaps they should be. Some simple remedies prove to be a power in the hands of some, while with others they seem to possess no curative virtues at all.

It is undoubtedly a fact that the actions of many drugs are modified, not only by different organisms, but by different climates and different surroundings and especially so when combined with other agents. It is my endeavor to-day to bring before you and, if possible, impress upon your minds some, at least, of the virtues I have found to be possessed by this cheap and efficient remedy namely "The Hyposulphite of Soda." This preparation is formed by boiling the filtered solution of Sulphite of Soda with Sulphur when after filtration and concentration the crystals are deposited in colorless transparent prisims or plates. It is odorless and has a cooling and somewhat bitter taste, is neutral or faintly alkaline in reaction and is soluble in 1.5 parts of water. Its main action is as an antiseptic but it is also a strong deodorizer, alterative and insecticide.

In the stomach it gives off sulphurous acid which, from its preparation, it resembles. It destroys bacteria and septic germs, arrests fermentations and removes offensive smells. It is much used in photography and forms an important part in some officinal tests.

* Read before the Ohio State Veterinary Medical Association:—

Among the first cases in which I used this salt with marked success was in the treatment of a valuable stallion, but recently brought from California and not yet acclimated.

Soon after arrival many parts of the body especially those covered by the harness, became covered by eruptions, and by their intense itching caused the animal to rub and produce a raw surface, and he could with difficulty be prevented from irritating it still more ; a peculiar black discharge issued from the anus continuously and trickled down the thighs, and so on to the ground ; his tail had become full of it and seemed from its tary consistency to defy thorough cleansing ; a peculiarly bad odor accompanied the discharges. His appetite was capricious.

His food and surroundings were of the cleanest and best, and the general attention bestowed upon him was befitting a $10,000 stallion. After thorough examination I put him on Hyposulphite of Soda, ℥ss. doses, three times a day with a very small quantity of gentian; eighteen powders, six days treatment, sufficed to cleanse and cool his blood, entirely allaying the skin irritation and regulate the bowel secretions to almost perfection.

In one year from that time he had a slight similar discharge from the bowels which was entirely overcome by one-half dozen powders of the same preparation.

During the past summer I have treated skin troubles whether dependent upon constitutional or local causes, almost entirely with this preparation of soda, and with universal good success. One case deserves mention especially.

A young pacing horse with considerable speed had been fed pretty high, to fit him for a good showing at the Fall sales at which he was entered. Unfortunately without much regular exercise he was given a long drive on a hot day. And the next morning I was called and found him one mass of pimples from the tips of his ears to his hoofs. In a few days he was as hairless as a Mexican dog, the skin scaling off and leaving an irritable itchy surface, which he rubbed and bit and actually tore with his teeth at every opportunity.

Solutions of Hyposulphite were applied externally and following a physic ℥j dose four times a day given internally. Other preparations were used for a day at a time externally, but none seemed to allay the irritation as well as the soda. With a cooling diet and an occasional stimulant for the kidneys, thirty days found him again on the road in fair flesh, good spirits and covered with a short, thick glossy coat.

Other cases of eruptions of less consequence—some affecting the larger part of the body, others only local have been successfully combatted and the animal kept at his work.

In speaking of this medicine Finlay Dun, in his excellent work, gives several interesting experiments, showing its power as a poison to fungi and other low organic forms, and many have been made mostly as recorded with the sulphite, showing its power to control septic conditions.

Dogs previously prepared for several days with small doses of Hyposulphite, have withstood injections of fœtid pus, and even survived the injection of the purulent discharge from a glandered horse.

It is in the treatment of Septicæmia and all putrid conditions of the system that I think this agent deserves a thorough trial. About one year ago I was called to see a promising colt, the right side of whose face was terribly swollen, from eight or ten openings issued a fœtid putrid discharge, somewhat dark in color and mixed with streaks of blood. The skin seemed to a large extent to be underrun and I had to open several pockets to get a drainage.

There were several openings into the mouth and the animal could scarcely eat even soft food. On his back just forward of the hip on the right side was a large swelling discharging from its one opening a fluid identical to all appearances with the other.

The pulse 82 and wiry, temperature 104½. I concluded that here we had a serious case, but could not give the direct cause, unless it originated with the teeth, which were quite sharp opposite the internal openings. I dressed the teeth and thoroughly cleansed the abscesses, using a Bichloride solution, 1 to 1,000. And left a solution for future injection of 1 to 5,000. I administered ℥j of Hyposulphite of Soda and left instructions that he should get ℥ss. every four hours in solution.

I saw my patient again in two days, pulse 60, temperature 103, eating more and feeling apparently better. The abscesses showed a more healthy discharge and were not inclined to spread. I continued the medicine for some days reducing it in quantity. My patient made a rapid recovery.

The owner of the colt is one of the leading physicians of Cleveland, and he took occasion soon after to call my attention more particularly to this remedy which was entirely new to him. Two weeks ago I had a long talk with him again and he was very enthusiastic over his success with it saying, "It had worked like a charm in every case." And he proceeded to call my attention to numerous cures he had effected with it, one or two of which are particularly worthy of mention.

From a scratch, or small injury a young man's hand and arm had become swollen almost out of resemblance to its original shape, the line of lymphatics to the shoulder were terribly enlarged and the hand and lower arm had assumed a leadened hue, which with the constitutional symptoms presented, but fair that an amputation of it might soon be necessary to save his life. However, it was resolved to use the Hyposulphite and note its effect if any, gr. xv. were administered every four hours and by the following day, a perceptible change for the better could be seen, and on the second day considerable hope was given that the arm would be saved. The fourth found him to all appearances out of danger, and he rapidly made a complete recovery.

Another, still more serious, was the case of a man whose foot had been crushed down at the instep, by the wheel of a heavy wagon, and in hopes of saving it amputation had been delayed until, almost before they realized the fact, it had become putrid. The limb was terribly swollen and pressure upon it even as high as the thigh would cause a fœtid discharge to issue from the opening below. After amputating just above the ankle, two large pus cavities were tapped above the knee and three below to get sufficient drainage for the purulent matter that was fast accumulating. Everyone who saw it thought there was little chance for the remainder of the leg or the patient's life, as he was much wasted and very weak. However, my friend, who was called in consultation, persuaded them to rely on the soda, which they did with very gratifying results, saving the patient's life and the remainder of the leg. In numerous cases of wounds large and small, where there was much discharge, I have used this agent and cannot compute the good it has done me. In the results of a runaway accident one horse had a wagon tongue thrust into his neck just beneath the third cervical, passing through to the skin of the opposite side and in his efforts to escape, muscles, trachea, œsophagus, arteries, and veins were stripped of their attachments and hung dangling like so many sections of slack-rope, one wing of the atlas was fractured and a large pocket opened below along the lower surface of the vertebra and behind the inferior cervical muscles. The depression of the head partially drained the several large pockets, and this case was treated successfully in a short time, by local applications of Bichloride and white solutions, and the Hyposulphite internally, there was scarcely any fœter at any time.

I could call your attention to other cases just as gratifying to me, but I wish to name another condition for which I have found my subject beneficial.

In the treatment of gastric and bowel troubles associated with fermentations, we find different gases generated in various proportions, according to the cause, time elapsed, and surrounding conditions. In cases apparently similar, I have found it necessary to use entirely different agents to gain my desired result. In the use of the Hyposulphite for these conditions I believe it to have been an essential adjunct in every case. Associated with the process of fermentation and the formation of gases there is always absorption to a greater or less extent of these poisonous products into the blood, and in many cases this is the sole cause of death. In the Hyposulphite we have one of the greatest antiseptics and blood purifiers. Thus, acting at the same time, to prevent absorption and to destroy the already absorbed, while striking directly at the cause, it is very effective in arresting the fermentation.

And again, in azoturia, although my experience is here limited, I am becoming more and more convinced, that it is most worthy of a thorough trial. Undoubtedly the whole line of symptoms in this disease is caused by the actions of poisonous products upon the nerves and their centers. Thus, directly through the condition of the blood, we are sure to get our several results.

Urea, is one of the natural constituents of urine excreted from the blood by the kidneys. In this disease immense quantities of it are excreted, showing an unnatural action of the kidneys or an inestimable quantity of it in the blood, which from its nitrogenous character is without doubt.

The Hyposulphite of Soda, acts directly to diminish urea and increase uric acid, the sulphates, sugar and other non-nitrogenous constituents, a condition certainly necessary to the recovery of the patient.

I have used it in several cases all of which have recovered. Two of the number were prostrated and could not rise and one that was placed in slings could not bear any weight on one hind leg for five days. Although standing his urine had to be drawn during the first three days. Considering the scarcity of my patients that have lived after being prostrated with azoturia and subjected to my previous treatment, I feel that my new departure deserves further trial which I shall certainly give it at my first opportunity.

I wish once more to trespass upon your patience, by drawing again from my memoranda of experience. Two cases of abortion—the first occuring about one week before I was called—was informed the animal appeared well for two or three days then began to be uneasy, ate little and gradually grew worse until at the present she ate nothing, was continually uneasy ; respiration 36, pulse 80, temperature 105° ; a fœtid discharge issued from the vulva and the abdomen had a tucked-up appearance, she strained at short intervals passing a colored discharge.

I found the vagina and uterus much swollen, the latter containing a small quantity of putrid matter. I cleansed the parts as thoroughly as I could although the effort caused much irritation. And being a little afraid to risk the Hyposulphite alone, I combined with it fair sized doses of aconite ; after two or three days of uncertainty she began to make a slow recovery which proved permanent.

The other, not as serious as I would have liked, I was called to attend within an hour after her untimely delivery, found her not much changed from the normal in pulse and temperature, inclined to eat, yet a little uneasy, and to all appearances the abortion was a complete one, I gave her a light opiate and waited developments. The next day she was more uneasy, lying down considerably, eating little, respiration 24, pulse 72, temperature 104½ and quite a profuse discharge from the vulva. I attended to her general comfort more especially, left instructions in regard to diet, administered ℥j of Hyposulphite in solution, and left similar doses to be given every four hours.

The next day, respiration 16, pulse 48, temperature 103°. The third day, respiration 14, pulse 36, temperature 100°. Complete recovery followed.

In conclusion, my experience with this agent has been highly satisfactory to me, and I could name many more cases where I attribute the sole cause of recovery to it. Let me urge all who have not given it a thorough trial to do so, and from those to whom it is familiar, I should be pleased to hear the results of their experiments.

